# Biochemical Changes in the Serum of Patients with Chronic Toxigenic Mold Exposures: A Risk Factor for Multiple Renal Dysfunctions

**DOI:** 10.1100/tsw.2003.92

**Published:** 2003-11-03

**Authors:** Ebere Anyanwu, Andrew W. Campbell, Aristo Vojdani, John E. Ehiri, Akpan I. Akpan

**Affiliations:** Neurosciences Research, Cahers Inc., Conroe, TX, USA

**Keywords:** chronic mycosis, renal disorder, toxic molds, analysis, public health, United States

## Abstract

This paper analyzes and presents the biochemical abnormalities in the sera of patients presenting with chronic mycosis in order to investigate the relationship with the risks of multiple renal disorders. The study population (n = 10) consisted of six females and four males (mean age 36.3 years) exposed by toxic molds in their homes and offices for an average of 2.8 years. The control group comprised ten people, five males and five females (mean age 35.9 years) without any known exposures to toxic molds. Blood samples were obtained from both the patients and the controls and were processed using specific biochemical methods that included enzyme-linked immunoabsorbent assay (ELISA). There were biochemical abnormal concentrations in creatinine, uric acid, phosphorus, alkaline phosphotase, cholesterol, HDH, SGOT/AST, segmented neutrophils, lymphocytes, total T3, IgG and IgA immunoglobulins with significant differences between patients and controls. These abnormalities were consistent with multiple renal disorders. The major complaints of the mycosis patients were headaches, pulmonary symptoms, allergic reactions, memory loss, skin rashes, blurred vision symptoms, fatigue, and runny nose. These findings were depictive of a strong association of chronic mycosis with abnormal renal indicators. It was concluded that, although this research was a pilot investigation, based on the overall results, people exposed to chronic indoor environmental toxic molds were at risk of multiple renal complications.

## INTRODUCTION

There is increasing concern about adverse health effects of indoor environmental toxic mold contamination on residents and workers whose homes and offices have been water-damaged by floods, dampness, and moisture.

These conditions are conducive to toxic mold growth. Molds readily enter indoor environments by circulating through doorways, windows, heating, ventilation systems, and air conditioning systems. The most common indoor toxic molds are *Cladosporium, Penicillium, Aspergillus,* and *Alternaria* species[1,2,3]. All toxic molds have the propensity to produce extremely potent toxins called mycotoxins[4,5,6], which are lipid-soluble and readily absorbed by the intestinal lining, airways, and skin. In the last 2 years, the complaints against toxic mold infection have increased, especially in those patients whose homes and offices in the Woodlands areas of Texas were contaminated by toxic molds.

In our experience, most of these patients complained of severe headaches, fatigue, and renal disturbances. The leading primary renal diseases are glomerulonephritis, pyelonephritis, and diabetes[7]. It is known that certain drugs such as Omeprazole are known to induce renal disorders. Omeprazole is a proton pump inhibitor that is used commonly in the treatment of acid-peptic disorders. Although Omeprazole is generally tolerated, serious adverse effects such as renal failure with a rising serum creatinine concentration have been reported[7]. Typical laboratory features included hematuria, proteinuria, pyuria, eosinophilia, and anemia[8]. A recent study[9] found that hemolytic-uremic syndrome was a thrombotic complication of *Escherichia coli* (O157:H7) infection. In the hemolytic-uremic syndrome, thrombin generation (probably due to accelerated thrombogenesis) and inhibition of fibrinolysis precede renal injury and may be the cause of such injury.

The main aim of this research was to carry out a pilot investigation to determine the renal disorder–related biochemical abnormalities in the sera of patients presenting with chronic mycosis and assess the outcomes in order to establish the relationship between chronic mycosis and the risk of multiple renal disorders.

## METHODS

The study population in this pilot study (n = 10) consisted of six female and four male (mean age 36.3 years) participants. Those that were exposed to toxic molds in their homes (infection site) have lived in them for an average of 6 years. Those that were occupationally exposed to toxic mold worked in their offices (infection site) for an average of 2.8 years. Data from the control group comprising five males and five females (mean age 35.9 years), but without any known exposure to toxic molds, were extracted from the Immunosciences Lababoratory database (Beverly Hills, CA). Exclusion criteria were based on “no known history of previous or present renal disease or diabetes.” A specially designed health questionnaire that reflected the demographics (sex, age, previous and present illnesses) of the patients and controls was dispensed. Independent laboratories (Armstrong Forensic Laboratories, Arlington, TX) carried out the tests on toxic mold contamination of the patients’ indoor environments using mycological and microscopic presence of mycohyphal projections in the matrix samples. The indoor toxic mold contamination reports were positive for different types of toxic molds including *Stachybotrys chartarum*, *Aspergillus*, and *Penicillium* species. Blood samples were obtained from both the patients and the controls, processed, and the serum analyzed using immunochemical techniques including enzyme-linked immunoabsorbent assay (ELISA) and Western blot procedures. All immunologic chemicals and reagents were purchased from Sigma Chemicals.

### Statistical Analysis

Descriptive statistics were conducted to describe the study population. A correlation matrix was generated using the primary variables of interest, which included fatigue, memory loss, runny nose, sleep disturbances, and allergic reactions. The association between biochemical changes in the patients and control with risk factors, and rate ratios of abnormalities were calculated by multiple Poisson regressions using SAS procedure GENMOD[10].

## RESULTS

There were strong associations of chronic mycosis with abnormal renal indicators and significant differences between patients and controls. The major complaints of the mycosis patients were headaches, pulmonary symptoms, allergic reactions, memory loss, skin rashes, blurred vision symptoms, fatigue, and runny nose. These effects occurred more in males. The indoor environmental contamination by toxic molds correlated well with the outcomes of the laboratory examinations of the patients' sera. Results from the laboratory analysis are presented in Table 1.

**TABLE 1**
**Abnormal Biochemical Changes in Patients with Chronic Mycosis and the Normal Controls**

| Test Parameters | Patient Samples (n = 10) | Control Samples (n = 10) | Mean Difference | Std Error Difference (df = 18; t = 2.10) | 95% Confidence Interval |
|---|---|---|---|---|---|
| Creatinine | 0.372 +/– 0.281 | 0.894 +/– 0.351 | –0.877 | 0.142 | 0.308–0.551 |
| Uric acid | 3.23 +/– 0.109 | 5.60 +/– 0.437 | 0.550 | 0.142 | 0.428–0.778 |
| Phosphorus | 7.45 +/– 0.466 | 3.64 +/– 0.216 | 0.714 | 0.699 | 1.76–2.37 |
| Cholesterol | 106 +/– 0.0354 | 188 +/– 0.0992 | 0.574 | 0.333 | 0.525–0.604 |
| LDH | 414 +/– 0.452 | 168 +/– 0.263 | 0.903 | 0.845 | 2.07–2.95 |
| SGOT/AST | 59.1 +/ 0.0992 | 25.7 +/– 0.272 | 0.832 | 0.915 | 1.90–2.78 |
| Alk. Phos. | 225 +/– 0.0557 | 59.5 +/– 0.841 | 1.33 | 0.267 | 2.16–6.62 |
| Chol/HDL | 2.28 +/– 0.101 | 5.68 +/– 0.207 | –0.910 | 0.0729 | 0.345–0.469 |
| Segmented neutrophils | 22.6 +/– 0.125 | 60.6 +/– 0.133 | –0.985 | 0.0577 | 0.331–0.422 |
| Lymphocytes | 67.1 +/– 0.949 | 29.4 +/– 0.249 | –0.825 | 0.0843 | 0.367–0.523 |
| Total T3 | 227 +/– 0.0458 | 129 +/– 0.331 | 0.564 | 0.106 | 1.41–2.19 |
| IgG Immunoglobulins | 472 +/– 0.0841 | 1039 +/– 0.263 | –0.789 | 0.0874 | 0.378–0.546 |
| IgA Immunoglobulins | 34.7 +/– 2.4 | 221 +/– 0.143 | –0.186 | 45.1 | –0.281 to –0.915 |

There are significant differences between the chronic mycosis patients and the normal controls. Observe that these changes are consistent with those found in patients with renal dysfunctions.

Serum creatinine concentration was very low (0.372 mg/dL +/– 0.281) in mycosis patients compared to the controls (0.894 mg/dL +/– 0.351), with mean difference of –0.877 and standard error difference (df = 18; t = 2.10) of 0.142 (95% CI = 0.308–0.551). The serum uric acid level was low (3.23 mg/dL +/– 0.109) as against the controls (5.60 mg/dL +/– 0.437) with mean difference of 0.550 and a standard error difference (df = 18; t = 2.10) of 0.142 (95% CI = 0.428–0.778). There was a high increase in mean total T3 (227 ng/dL +/– 0.0458) compared to the controls (129 ng/dL +/– 0.331), mean difference being 0.564 with a standard error difference (df = 18; t = 2.10) of 0.106 (95% CI = 1.41–2.19). The mean phosphorus was also very high (7.45 mg/dL +/– 0.466) relative to the controls (3.64 mg/dL +/– 0.216) with a mean difference of 0.714 and a standard error difference (df = 18; t = 2.10) of 0.699 (95% CI = 1.76–2.37). The alkaline phosphotase was also very high (225 U/L +/– 0.0557) compared to the controls (59.5 U/L +/– 0.841) with a mean difference of 1.33, standard error difference (df = 18; t = 2.10) of 0.267 (95% CI = 2.16–6.62). Cholesterol level was low (106 mg/dL +/– 0.0354) compared to the controls (188 mg/dL +/– 0.0992). The mean difference was 0.574 with a standard error difference (df = 18; t = 2.10) of 0.333 (95% CI = 0.525–0.604). There was a high increase in HDL of 414 μg/L +/– 0.452 compared to the controls of 168 μg/L +/– 0.263. The mean difference was 0.903 with a standard error difference (df = 18; t = 2.10) of 0.845 (95% CI = 2.07–2.95). There was a high increase in SGOT/AST of 59.1 +/– 0.0992 in the patients compared to the controls of 25.7 +/– 0.272.

The means difference was 0.832 with a standard error difference (df = 18; t = 2.10) of 0.915 (95% CI = 1.90–2.78). The Chol/HDL ratio was (2.28 +/– 0.101) in the patients as against the controls of 5.68 +/– 0.207 with mean difference of –0.910 and a standard error difference (df = 18; t = 2.10) of 0.0729 (95% CI = 0.345–0.469). The segmented neutrophils were low (22.6% +/– 0.125) in the patients compared to the controls of 60.6% +/– 0.133 with mean difference of –0.985 and a standard error difference (df = 18; t = 2.10) of 0.0577 (95% CI = 0.331–0.422). A high increase in lymphocytes of 67.1% +/– 0.949 was observed compared to the controls of 29.4% +/– 0.249 with mean difference of –0.825 and a standard error difference (df = 18; t = 2.10) of 0.0843 (95% CI = 0.367–0.523). The IgG immunoglobulins concentration was low (472 mg/dL +/– 0.0841) compared to the controls of 1039 mg/dL +/– 0.263 with mean difference of –0.789 and a standard error difference (df = 18; t = 2.10) of 0.0874 (95% CI = 0.378–0.546). The IgA immunoglobulins level was also low (34.7 mg/dL +/– 2.4) compared to the control of 221 mg/dL +/– 0.143 with a mean difference of –0.186 and a standard error difference (df = 18; t = 2.10) of 45.1 (95% CI = –0.281 to –0.915).

## DISCUSSION

Indoor toxic mold exposure leads to adverse health effects that are collectively known as mycosis. Although some of the manifestations are still emerging, the renal-related biochemical changes have not been fully documented. In our research, we found biochemical changes consistent with renal dysfunctional symptomology. These changes included abnormal serum creatinine, uric acid, phosphorus, alkaline phosphotase, cholesterol, enzyme proteins, thyroids, immunoglobulins, and lymphocytes cells. Serum concentration of creatinine is relatively constant and somewhat greater in males than in females, i.e., 0.6–1.5 mg/dL (53–106 mmol/L) for males and 0.5–1.0 mg/dL (44–88 mmol/L) for females when specific analytical methods were used (true creatinine). Creatinine was associated with an increase in T3, especially in our male patients. No such abnormalities were seen in the control group. Our patients showed abnormal serum uric acid/hypouricemia. This represented a suppression of renal uric acid excretion probably as a result of lactate excess, produced by the oxidation of ethanol to acetaldehyde, which might have competitively inhibited renal tubular secretion leading to renal tubular acidosis. Renal tubular acidosis was defined as defective secretion of $H^+$ by the renal tubules in the presence of normal or near normal glomerular filtration rates (GFR). Either of the distal elements such as the collecting ducts or proximal tubules could have been responsible. In human subjects, the assessment of renal function and of its changes by interventions is limited to the measurement of GFR, renal blood flow, and the estimation of proteinuria. Serum creatinine concentration is a commonly used measure of renal function in clinical practice. Differences in creatinine production among subjects and over time in a single individual may occur because of changes in muscle mass[11].

Mild elevation of serum aspartate aminotransferase (AST) (glutamate oxaloacetate transaminase) levels has been reported in patients with pulmonary infarction, but not in chronic mycosis. This enzyme is elevated in diseases involving the tissues that are rich in it such as heart, liver, skeletal muscle, kidneys, brain, pancreases, spleen, lungs, and serum[12]. The incidence of increased values in humans is low, the degree of abnormality slight, and the rise delayed for 3–5 days after onset of pain[13]. Infusion of phosphate increases distal $Na^+$ delivery, allowing for greater exchange with hydrogen. Because doubly charged anions are not readily absorbed, they capture hydrogen and prevent the back-leakage into the blood seen with gradient defects. Normally, hypouricemia is caused by renal tubular reabsorption defects, either congenital (as in the Fanconi syndrome and Wilson's disease) or acquired (particularly through toxic damage). Under these conditions, increased urinary loss of urate and low plasma levels can be caused. Additionally, the condition could be associated with malignant disorders such as multiple myeloma, auditory neuroma consistent with abnormal parathyroid hormone (PTH) secretion, and low serum levels of phosphates and 1,25-dihydroxyvitamin D in uremic patients than in the controls[14]. Low levels of PTH were observed in our study. Decreases in PTH levels have an "anabolic-like" effect on bones with a low-turnover lesion in this animal model of chronic renal insufficiency[15].

Phosphorus comprises about 1% of the total body weight of humans and 85% of it is stored in the bone in the form of hydroxyapatite crystal; 14% is in the soft tissues in the form of energy-storing bonds with nucleotides (ATP, GTP), nucleic acids in chromosomes and ribosomes, 2,3-DPG in the red blood cells, and phospholipids in the cells' membranes[16]. High phosphorus concentration was found among our patients and that was probably indicative of renal dystrophy, hypothyroidism, pseudohypothyroidism, or renal tubular acidosis. Phosphate balance is maintained by multiple systems. The gut is responsible for the absorption of two-thirds of the 4–30 mg/kg/day of phosphate intake. Absorption sites are all along the gut; the jejunum is the most active site in humans. The kidney filters 90% of the plasma phosphate and reabsorbs it in the tubuli. In states of hypophosphatemia, the kidney can reabsorb the filtered phosphates very efficiently, reducing the amount excreted in the urine virtually to zero[16]. These findings are indicative of multiple systemic involvements in phosphate balance. Therefore, our result findings seemed to indicate that the mycotoxins interfered with the complex control of phosphate homeostasis, leading to various clinical conditions such as gastrointestinal malabsorption and increased urinary losses due to tubular dysfunction. High phosphorus intakes for 10 days are shown to reduce the plasma calcitriol levels by 70% and several lines of evidence indicated that prolonged high phosphorus intake may impair the usual homeostatic mechanisms that come into play when dietary calcium is limited. This could in turn affect the bone mass[17]. Alkaline phosphotase, on the other hand, is involved in primary hyperthyroidism, renal ostedystrophy, vitamin deficiency, and renal tubular acidosis. Low blood cholesterol and DHL levels of <200 mg/dL have long been appreciated as desirable in terms of cardiovascular risk. Individuals with high triglycerides and low HDL cholesterol, but not low-density lipoprotein cholesterol, have an increased risk of renal dysfunction[18]. Low segmented neutrophils may represent deficiencies of humoral factors including antibodies, components of complements, or cellular abnormalities including contractile protein dysfunction, enzyme deficiencies, or may be caused by defects in motility, phagocytosis, or mycotoxic activities. Stoev et al.[19] induced mycotoxic nephropathy in 18 young pigs by diets contaminated with strains of *Aspergillus ochraceus* containing ochratoxin A (OTA) and penicillic acid (PA) at levels corresponding to those naturally encountered in animal feeds in Bulgaria. A mottled surface of the kidneys was only seen in pigs exposed to a moldy diet containing 180 ppb OTA for 3 months, but microscopic lesions, as well as changes in various hematological and biochemical parameters, were observed in all groups exposed to the same moldy diet containing only 90 or 180 ppb OTA. Histological examination showed two types of change: degenerative changes affecting the epithelial cells of the proximal tubules, which predominated at the initial stage, and proliferative changes in the interstitium, which predominated at the later stage of the disease. Telangiectasis and lymph stasis were also seen, as well as degenerative changes in the capillary endothelium. The characteristic renal lesions were similar to those observed in spontaneous cases of mycotoxic porcine nephropathy in Bulgaria, but they were a little different from the classic Danish porcine nephropathy. The enhanced toxicity of OTA in our study may be due to a synergistic effect between OTA and PA or to some other unknown metabolites produced by the same ochratoxinogenic strains of *A. ochraceus*[19].

Patients with acidosis show abnormal uric acid and high serum triglycerides, low serum HDL cholesterol, but normal total cholesterol, plasma valine, leucine, and isoleucine than control values ($p < 0.01$) and these correlated with serum creatinine[20]. Stoev et al.[21] found renal changes were characterized by impairment of proximal tubular function (indicated by an increased urinary excretion of glucose and protein). The concentration of urea, creatinine, and glucose in the blood was increased, whereas the serum protein and cholesterol were decreased in animal models with 10–20% and 50–60% frequency of nephropathy. This condition may result in the presence of granular casts and necrotic renal tubular cells[21]. IgA in the serum is typically responsible for this neutralization, suggesting a unique role for serum IgA in response to toxic infection that extends to human systems especially, the intestinal mucosa[22]. The relationship of naturally occurring antitoxin antibodies to the clinical course of mycotic infection is not clear. However, patients in our Center with chronic toxic molds infections and low levels of IgG to the mycotoxin have shown clinical responses following intravenous therapy with immune globulin. Low IgA is associated with a condition known as IgA nephropathy (IgAN). It is manifested by glomerular, vascular, and interstitial fibrosis indices and can show the degree of tubular atrophy or the diffuse extent of mesangioproliferative glomerulonephritis [23,24]. Low IgG may be a potential contributor to development of renal failure, and its presence in tubular fluid may contribute to the hypercalciuria, interstitial fibrosis, and the progressive renal failure of Fanconi syndromes[25]. Both IgA and IgG levels increased in older adults and different immunoglobulins seemed to be considerably correlated, especially in IgG type antibody and serum IgG level[26]. A high increase in lymphocyte levels was observed, mostly in patients with auditory mycotic neuroma. This observation was consistent with Opat et al.[27], who found a significant increase of IgA (3.73 g/L on the average) in patients with malignant lymphoma compared to the controls (mean 2.31 g/L).

## CONCLUSIONS

The adverse effects of chronic indoor toxic mold exposures are emerging as one of the public health concerns, because of the increased debilitating health complications and costs of treating them. Most of the patients in this small pilot study complained of severe headaches, fatigue, loss of memory, and renal disturbances. The primary renal disturbances indicated were glomerulonephritis and pyelonephritis. Although renal disorders are associated with different etiologies, however, none are more supported than the pieces of evidence from the biochemical changes in patients with chronic toxic mycosis and although this research was a pilot investigation, the overall results seemed to suggest that people exposed to chronic indoor environmental toxic molds are at risk of multiple renal complications. Further studies on a larger scale are encouraged.

## BIOSKETCHES

**Ebere C. Anyanwu, Ph.D.,** is a Clinical Neurophysiologist and Director of Cahers Neurosciences Research Inc., Houston, TX and also a member of the Biomonitor Research Group, Department of Chemistry, Texas Southern University, Houston. He is also an Adjunct Professor of Anatomy and Physiology at the North Harris and Montgomery Colleges in Houston and The Woodlands, respectively.

**Andrew W. Campbell**, **M.D.** is a Medical Scientist in Immunotoxicology, Medical Director and Chief Executive of the Center for Immune, Environmental and Toxic Disorders, Houston, TX. E-Mail: md@immunotoxicology.com

**Aristo Vojdani**, **Ph.D.,** is a professor of immunochemistry and the Chief Executive of the Immunosciences Laboratory, Inc., Beverly Hills, CA. Aristo Vojdani is also Board Certified in Medical Laboratory Technology. E-mail: immunsci@ix.netcom.com

**John E. Ehiri**, **Ph.D,** is an Assistant Professor in the Department of Maternal and Child Health, School of Public Health, University of Alabama, Birmingham. E-mail: jehiri@uab.edu

**Akpan I. Akpan, Ph.D.,** is Associate Professor in the School of Public Affairs, Urban Planning and Environmental Policy and Management, Texas Southern University, Houston. Formerly Professor of Urban Planning and Environmental Sciences in Nigeria for many years. E-mail: akpanakpan2002@yahoo.com
